# Testing the Impact of Familiarity with Health Benefits Information on Dietary Supplement Choice in Pregnancy: An Online Choice Experiment

**DOI:** 10.3390/nu14091707

**Published:** 2022-04-20

**Authors:** Lenka Malek, Wendy J. Umberger, Shao-Jia Zhou, Elisabeth Huynh, Maria Makrides

**Affiliations:** 1 Centre for Global Food and Resources, Faculty of Arts, Business, Law and Economics, The University of Adelaide, Level 6 NEXUS 10 Tower, 10 Pulteney Street, Adelaide, SA 5005, Australia; wendy.umberger@adelaide.edu.au; 2 School of Agriculture, Food and Wine, Waite Campus, The University of Adelaide, PMB 1, Adelaide, SA 5064, Australia; jo.zhou@adelaide.edu.au (S.-J.Z.); maria.makrides@sahmri.com (M.M.); 3 Robinson Research Institute, The University of Adelaide, Adelaide, SA 5005, Australia; 4 Department of Health Services Research and Policy, National Centre for Epidemiology and Population Health, ANU College of Health and Medicine, Australian National University, Acton, ACT 2006, Australia; elisabeth.huynh@anu.edu.au; 5 Women and Kids, South Australian Health and Medical Research Institute, Women’s and Children’s Hospital, 72 King William Road, North Adelaide, SA 5006, Australia

**Keywords:** dietary supplements, folic acid, iodine, nutrition knowledge, discrete choice experiment, health benefits, awareness, pregnancy, nutrient recommendations, food choice

## Abstract

To help meet the increased requirements for critical nutrients during and around pregnancy, supplementation with essential nutrients is recommended. This study aims to determine how the previous awareness of nutrient health benefits and/or the provision of this information influences the importance placed on nutrients (folate, iodine, omega-3 fatty acids, and vitamin D) when choosing between dietary supplement products for pregnancy. Discrete choice experiment data were collected as part of a cross-sectional online survey administered to 857 pregnant women living in Australia. Four segments of women were identified that differ in their preference criteria when choosing among dietary supplement products for pregnancy. When choosing between products, the reinforcement of perceived health benefits (i.e., showing information on health benefits to those already aware of the benefits) was most effective at increasing the importance of folate (in all segments) and iodine (in two segments, 63% of the sample). Neither prior awareness of health benefits alone nor information provided at the point-of-purchase without prior awareness were enough to increase the importance of folate. Our findings suggest a need for simultaneous strategies that (1) provide information on health benefits before purchase and (2) ensure that information on health benefits is available at the point-of-purchase.

## 1. Introduction

The prevention of chronic disease and related healthcare expenditures starts before birth. According to a large body of scientific literature, maternal nutrition from preconception to lactation is associated with infants’ growth, cognitive development, and lifetime risk of developing chronic disease [1]. To help ensure that women meet the increased requirements for critical nutrients during and around pregnancy, supplementation with essential nutrients is recommended [2,3]. Supplementation with folic acid is recommended globally; 400 µg per day is recommended in the one month before conception and in the first trimester of pregnancy for the prevention of neural tube defects (NTDs). Supplementation with iodine is also recommended in Australia; 150 µg per day is recommended during the periconceptual period, pregnancy, and lactation to reduce the risk of iodine deficiency, which has been associated with impaired fetal growth and cognitive development. Although the mandatory fortification of staple foods with folic acid and iodine (introduced in 2009) has reduced the incidence of folic acid deficiency among childbearing-aged women (and the incidence of NTDs has decreased by 14% [4]), a relatively high prevalence of folic acid insufficiency remains among women of childbearing age (39% in 2010) [5]. Likewise, evidence suggests that pregnant women remain at risk of iodine deficiency without iodine supplementation [4,6,7].

Supplement use during pregnancy is common in developed countries, yet few women adhere to their country-specific supplement recommendations [8,9,10]. Adherence to food-based dietary guidelines during pregnancy is also poor [11]. This makes supplementation with key nutrients even more critical. While other factors may act as barriers to adhering to supplement recommendations (e.g., forgetting to take supplements) [12], purchasing a product that provides the advised daily dosage of nutrients is key to enabling supplement use in line with recommendations.

Food choices and the selection of dietary supplement products can be influenced by an extensive and dynamic range of factors [13,14,15]. These can include the attributes or characteristics of the individual/decision maker (e.g., socio-demographic, physiological, psychosocial, and cognitive variables) and of the food/supplement product under consideration (e.g., food type, price, nutritional content, flavour, brand, and other sensory properties and food labeling information), as well as other contextual factors in the decision-making scenario (e.g., the purpose for which product is being purchased, the availability of products, previous experience, advice/recommendations received, etc.) [16,17].

An individual’s nutrition knowledge can influence food product preferences and, ultimately, product choice. Findings from a recent review suggest a positive association between nutrition knowledge and food label use [18]. Specifically, nutrition knowledge can influence understanding, perceptions, and, subsequently, the use of point-of-purchase (POP) nutrition information and health claims [18,19]. The effect of POP ‘on-package’ nutrition information on food product choice varies, with on-package nutrition information more commonly found to reduce preferences for less healthy products than to increase preferences for healthier products [20,21].

Preference elicitation studies that focus on fortified food/beverage products and/or supplement tablets typically show that providing information on a product’s health benefits or a nutrient/ingredient can increase product preferences [22,23,24,25,26]. However, the effect that information about health benefits has on product choice can differ across consumer segments, such that not all consumers are influenced [25,26]. Of those who are, some are more influenced by the information than by the nutrients/ingredients providing the health benefit [25]. Thus, the existing literature shows that nutrition knowledge and information on health benefits can play an important role in using nutrition information and in the choice process; it also shows that effects vary between individuals (i.e., across consumer segments).

What remains to be investigated in the food choice and health literature is whether prior awareness of the health benefits of nutrients (herein referred to as ‘prior knowledge’) or the provision of this information at the POP has a greater impact on the use of nutrition information when making food or dietary supplement choices. Moreover, the impact of health benefits information on product preferences has not previously been examined in the context of choosing a dietary supplement product for use during pregnancy, a critical life stage.

Elsewhere, we showed that pregnant women place relatively little importance on nutrient levels when choosing between different nutritionally-fortified food and beverage products and supplement tablets [27]. Here, we build on these findings by investigating the effect of health benefits information on the relative importance of nutrient levels when choosing between dietary supplement products for pregnancy. Specific study objectives were to determine whether and how a previous awareness of nutrient health benefits and/or the provision of this information when making choices (i.e., at POP) influence the effect of nutrients on the choice decision in consumer segments with different product preferences. In line with the literature, we hypothesised that the largest positive increase in preferences for products containing key nutrients would be observed among women who both have previous awareness of the health benefits and are shown this information when choosing between products, thus reinforcing health benefits information.

## 2. Materials and Methods

### 2.1. Discrete Choice Experiment (DCE)

We collected DCE data as part of an extensive cross-sectional web-based survey examining nutrition knowledge, attitudes, and practices during pregnancy. DCEs can be used to determine individuals’ preferences for products and product attributes for both on-market and hypothetical products [28] and have been shown to produce reliable predictions of health-related behaviours [29]. This method typically requires individuals to choose their most preferred product from a subset of two or more products. Each product is described by a unique and statistically determined combination of attribute levels (e.g., cost, brand, health benefit, etc.). The choice experiment in our study was hypothetical in nature and assessed preferences for three alternative forms of dietary supplement products: tablets (on the Australian pregnancy supplement market), fortified food (on the pregnancy supplement market in the form of candy only), and fortified beverages (not on the pregnancy supplement market).

### 2.2. Attributes and Attribute Levels

The attributes and attribute levels included in the DCE are shown in Table 1. Our selection of attributes was informed by reviewing the relevant literature, by the supplement recommendations of Australian health authorities [2,3], and by marketplace observation. We included nutrients for which there exist population-wide supplement recommendations (folate and iodine) in addition to nutrients that are not recommended on a population basis (i.e., for all pregnant women) but are commonly found in pregnancy/prenatal supplements in Australia (omega-3 and vitamin D). Further details regarding the attributes and attribute levels are provided in [9].

### 2.3. Experimental Design and Information Treatments

We used an orthogonal main effects plan (OMEP) experimental (within-subject) design to determine the combinations of nine attributes and their levels for the choice scenarios considered in this study. The 162 resulting choice sets were blocked into nine blocks, and each respondent was randomly allocated to one of the nine blocks and completed 18 choice scenarios (more details provided in [27]).

To explore the effect of health benefits information on choices, we used a between-subject design to randomly assign respondents to one of four specific information conditions for each nutrient (e.g., whether the benefits of folate are perceived and/or shown). For each nutrient, respondents were stratified according to prior awareness of the health benefits, as established earlier in the survey, and were randomly assigned to being shown or not shown the health benefits information. Using folate as an example, there were four different ways of allocating information regarding the benefits of folate: if the respondent *was not aware* of the benefits of folate, then they were either (1) *shown* or (2) *not shown* the health benefits information; and if the respondent *was aware* of the benefits of folate, they were either (3) *shown* or (4) *not shown* the health benefits information.

The choice experiment considered four nutrients, and the between-subject (2^J^) design ensured that each respondent saw a specific combination of health benefits information (information condition) from 2^4^ = 16 total possible options (see Table 2). Thus, respondents could be shown health benefits information for none of the four nutrients or for up to four nutrients. Each respondent saw their specific combination of health benefits information during the choice task (below the choice scenario), and this combination was fixed across all choice sets they completed. The health benefits shown for each nutrient are presented in Table 1.

### 2.4. Questionnaire

Before the choice task, respondents answered multiple-choice questions assessing their beliefs about the health benefits of folate, iodine, omega-3 fatty acids, and vitamin D (see Figure S1). Responses to these questions were used to randomly allocate respondents to each nutrient’s four possible information conditions. The 18 choice sets presented to participants each showed three labelled products: ‘Fortified food’, ‘Fortified drink’, and ‘Supplement tablet’. Within each choice set, participants were asked to (1) choose their most preferred product and (2) indicate if they would realistically purchase their chosen product. Choices were made in the context of the following scenario: ‘Imagine that you have just found out you are pregnant and you are shopping for a product to enhance your dietary intake during pregnancy’. An example of a choice set is provided in Figure S2.

In addition to collecting the DCE data, the questionnaire assessed a broad range of behavioural, psychosocial, knowledge, socio-demographic, and pregnancy-related variables that have been described previously [8,11].

### 2.5. Sample and Data Collection

The online survey was administered to a community-based sample of pregnant women aged ≥18 years and living in Australia. Women were recruited via two methods: (1) a reputable online panel provider (Pureprofile) supplied a national cohort, and (2) the researchers recruited a South Australian cohort through a large tertiary public maternity hospital. Data were collected between July and November 2013. Ethics approval was granted by the Women’s and Children’s Health Network Human Research Ethics Committee (HREC/13/WCHN/32) and the University of Adelaide Human Research Ethics Committee (H-2013–016). Further details of the study design and recruitment are reported in [11].

The analysis excludes 39 respondents (39/857 = 0.046) who always selected the same alternative (either the food, beverage, or tablet) in each choice set, reflecting non-trading behaviour. Thus, the present analysis uses data from the remaining 818 respondents.

### 2.6. Statistical Analysis

We estimated a latent class choice model using the intention to purchase the most preferred alternative as the dependent variable (0 = no, 1 = yes). Latent class choice models are a type of random utility model which estimates the probability of individuals choosing a specific alternative from a subset of alternatives and probabilistically assigns individuals with similar preferences to classes (herein termed ‘segments’) [30,31,32,33]. Further details of the utility and class-membership equations underlying the choice model estimation are reported in [27]. We modelled four interaction effects for each nutrient, which represent the nutrients’ impact on product choice under the four different information conditions. For a specific nutrient, the parameter estimate of each information condition represents the additional effect of the nutrient over and above its marginal effect given the health benefits information condition. We performed the choice model estimation in Latent Gold Syntax version 5.1 (Statistical Innovations, Inc. Belmont, MA, USA).

## 3. Results

In total, 857 pregnant women completed the survey (representing 56% of women who commenced the survey). No information is available on non-responders. However, the sample of 818 pregnant women (*n* = 432 from the national cohort and *n* = 386 from the South Australian cohort) on which the present analysis is based is nationally-representative of all women giving birth in Australia in 2012 with respect to maternal age, parity (proportion with no previous children), location, and country of birth (Australia vs. other) [34]. These data are reported in [27].

Table 3 shows the proportion of participants who associated each of the four nutrients (folate, iodine, omega-3 fatty acids, and vitamin D) with different health benefits. Overall, 75% of participants were aware that folate ‘Prevents neural tube defects such as spina bifida’; fewer (44%) were aware that iodine is ‘Important for baby’s brain development’; and over half were aware of the health benefits associated with omega-3 fatty acids and vitamin D. Based on these data, respondents were randomly assigned to an information condition for each nutrient. Table 2 shows the number of respondents allocated to each information condition.

We considered the optimal choice model to be one that identified four segments of pregnant women. We selected this model based on the minimum Bayesian Information Criterion (BIC) value (i.e., we tested models with up to six latent classes and observed the lowest BIC value with a four-class model; Table S1). Parameter estimates showing the contribution of each attribute level to the choice decision are presented in Table 4. The impact that each information condition has on the parameter estimate of each respective nutrient is shown in Table 2. The interaction effect shown in Table 2 can be added to the independent effect of the nutrient shown in Table 4 to show the total effect of each nutrient under the specific information condition.

Overall, the four segments reported here are consistent in size and have similar preference criteria to those reported and profiled (in terms of their behavioural, psychosocial, socio-demographic, and pregnancy-related characteristics) in [27]. This paper’s novel contribution lies in the demonstration of the effect of the nutrients on product choice under different information conditions.

### 3.1. Attribute Preferences of Each Segment

The four segments are briefly described below regarding the attributes that significantly influence their choice of dietary supplement products for pregnancy (Table 4) and the individual variables that differentiate each segment from the others (Table 5).

#### 3.1.1. Segment 1 (20%)

Segment 1 comprises women more likely to prefer beverage and food products over tablet products (Table 4). Levels of omega-3 fatty acids and vitamin D each significantly independently and positively influenced choice. In contrast, levels of folate and iodine only influenced choice when they were both present in the product. Women in this segment preferred products endorsed by the Commonwealth Scientific and Industrial Research Organisation (CSIRO) or the Dietitians Association of Australia (DAA). On average, women in this segment were younger than women in the other segments and were less likely to adhere to the iodine supplement recommendations than women in Segment 4 (Table 5).

#### 3.1.2. Segment 2 (22%)

Women in Segment 2 preferred food and beverage products over tablets and were more price-sensitive than other segments (Table 4). Omega-3 fatty acids and vitamin D were the only nutrients that positively and independently influenced product choice. Overall, women in this segment preferred products endorsed by the CSIRO and DAA and were averse to products with no endorsement or those endorsed by the NHMRC. On average, women in this segment were two years older than women in Segment 1 and two years younger than women in Segment 4 (Table 5). They were also less likely to be taking dietary supplements during their current pregnancy than women in Segment 3 and Segment 4.

#### 3.1.3. Segment 3 (43%)

Segment 3 is the largest segment. It comprises women who did not discriminate between product forms and placed greater importance on the levels of the four nutrients than all other segments (Table 4). Product endorsement by the CSIRO increased the appeal of foods and beverages, while endorsement by the NHMRC increased the appeal of tablets. Women in this segment were more likely to be nulliparous than women in Segments 2 and 4 (Table 5). They were also more likely to report taking supplements during pregnancy than women in Segment 1 and Segment 2.

#### 3.1.4. Segment 4 (15%)

Segment 4 is the smallest segment and comprises women who preferred supplement tablets over foods and beverages (Table 4). Vitamin D was the only nutrient that significantly independently and positively influenced product choice. On average, women in this segment were older than women in the other segments (Table 5).

### 3.2. The Effect of Information Conditions on Preferences for Specific Nutrients

Overall, being shown information had an additional positive effect on preferences for nutrients, while not being shown information had an additional negative effect on preferences for nutrients; however, these effects varied by nutrient, segment, and prior awareness of the information. The parameter estimates presented in Table 2 show how the interactions between information conditions and nutrients influence the effect that nutrient levels have on choice. For all segments, results show that the effect of folate levels on product choice depends on the information condition under which the choice was made. This was also the case for three of the four segments for iodine and for one and the same segment for omega-3 and vitamin D.

The results partially support our hypothesis that the largest positive increase in preferences for products containing key nutrients will be observed among women who have previous awareness of the health benefits and are shown this information when choosing between products. This was found to be the case for all four segments for folate, for two segments (Segments 1 and 3) for iodine, and just one segment (Segment 3) for omega-3 and vitamin D.

#### 3.2.1. Folate

Folate levels significantly independently influenced product choice in one segment only, with higher levels increasing product preferences (Segment 3: the main effect of every 100 μg of folate on choice was β = 0.255; Table 4). In the other segments, folate levels influenced product choice only under certain information conditions. Unlike the other nutrients, significant interactions between folate levels and information conditions were found in all four segments. Reinforcing health benefits information (i.e., showing information that women indicated prior awareness of) significantly increased preferences for products with higher folate levels in all segments. In all other cases where information conditions significantly influenced folate preferences, the effect was negative. For example, in Segment 3, the effect of every 100 μg of folate on choice was increased from β = 0.255 to β = 0.346 (obtained by summing 0.255 and 0.091) when women were previously aware of and were shown the health benefits of folate; the effect was reduced from β = 0.255 to β = 0.122 (obtained by subtracting 0.133 from 0.255) when women were not previously aware of and were not shown the health benefits of folate.

#### 3.2.2. Iodine

Higher iodine levels significantly independently (and positively) influenced product choice in Segment 3 only. Significant interactions between iodine levels and information conditions were found in three segments. Women’s preferences for products with higher iodine levels were significantly increased when reinforcing health benefits information in Segment 1 and Segment 3; and when showing health benefits information to women without prior awareness in Segment 2 and Segment 3. In contrast, women’s preferences for products with higher iodine levels were significantly reduced when known health benefits information was reinforced in Segment 2 and under the no information condition (not aware and not shown) in Segment 3.

#### 3.2.3. Omega-3

Higher omega-3 levels significantly independently and positively influenced product choice in all segments except Segment 4. Significant interactions with information conditions were found in Segment 3 only, which was also the segment most strongly influenced by omega-3 levels. Reinforcing perceived health benefits in Segment 3 further increased women’s preferences for products with higher omega-3 levels, while not showing information to women (irrespective of prior awareness) significantly reduced preferences.

#### 3.2.4. Vitamin D

Higher vitamin D levels significantly independently and positively influenced product choice in all four segments. As with omega-3, significant interactions between vitamin D levels and information conditions were seen in Segment 3 only. All information conditions significantly influenced preferences for higher vitamin-D levels, with an increase in preferences observed when showing health benefits information in choice sets (irrespective of prior awareness) and a reduction in preferences observed when not showing health benefits information in choice sets (irrespective of prior awareness).

## 4. Discussion

This is the first known study to experimentally manipulate the information conditions under which choices are made to determine whether and how health benefits information obtained prior to vs. at the point-of-purchase impacts the role of nutrient levels when choosing between different dietary supplement products. We do this in the context of pregnancy. We include in our investigation both nutrients for which there do (folate and iodine) and do not exist (omega-3 and vitamin D) population-wide supplement recommendations.

Our results reveal that reinforcing perceived health benefits information at the POP can increase women’s preferences for nutritional supplement products containing folate. We further demonstrate that neither prior awareness of health benefits alone nor information provided at the POP without prior awareness is enough to increase the appeal of folate-containing products. This suggests that folate levels are not important in the choice decision for most women unless health benefits information is reinforced at the POP.

It was a different story for iodine, with the reinforcement of health benefits information increasing iodine preferences in only two of the four segments (Segments 1 and 3, comprising 63% of the sample). In the remaining two segments, preferences for iodine were either not influenced by health benefits information (Segment 4) or were influenced in cases where health benefits information was shown (Segment 2). Showing information increased iodine preferences among women with no prior awareness and decreased iodine preferences among those with prior awareness. This decrease in iodine preferences is consistent with the negative (although not statistically significant) independent effect of iodine observed in that segment (Segment 2). The negative impact of POP information amongst those with prior awareness of iodine health benefits could be due to various factors. Compared to the recommendation for folic acid, the iodine supplement recommendation is more recent (introduced in 2012 vs. 1994). Previous research also suggests that supplementation with iodine is less often discussed during pre-and ante-natal consultations by HCP in Australia relative to the folate recommendation [8,35]. Therefore, women may be less familiar with and possibly more skeptical of the iodine recommendation; thus, weaker beliefs about the health benefits of iodine could explain this finding.

Further, consumers’ use of nutrition information on food labels generally depends on how useful they perceive the information to be [18,20]. In particular, supplementation decisions during pregnancy are influenced by perceptions of both the efficacy of supplementation and personal risk of/vulnerability to adverse pregnancy outcomes [12,36,37,38]. Thus, it is plausible that in cases where preferences for nutrients (folate and iodine-containing products) were not influenced or were negatively influenced by POP health benefits information, this could be due to women: not perceiving supplementation to be an effective risk-reduction strategy, not considering themselves to be at risk, or being skeptical of the information; all of these possibilities would be expected to impact the perceived usefulness of the health benefits information in the choice decision. Our study did not assess perceptions of: the usefulness of the health benefits information; personal vulnerability to adverse pregnancy outcomes (specifically, those referred to in the information conditions); maternal control in fetal health [39,40]; nor the ability of supplementation to reduce risk of adverse pregnancy outcomes. Thus, future studies seeking to understand the heterogeneous impact of health benefits information on product choice might consider assessing these factors. This could help increase understanding of why some women do not respond to iodine information, potentially leading to more effective strategies for enabling appropriate supplement choices.

Notably, our finding that not all consumer segments were influenced by health benefits information obtained before the survey or provided during the choice tasks is consistent with the broader literature [25,41,42,43]. For example, in their exploration of preference heterogeneity for a functional food product (inulin-enriched bread), Bitzsios et al. [25] showed that only some consumer segments were influenced by health benefits information. Similar findings have been reported when examining nutrition awareness. While nutrition awareness tends to be higher among pregnant women than among women who are not trying to conceive, there exists heterogeneity in nutrition awareness among pregnant women [41,42]; with some women ‘going all the way’ and some ‘taking the flexible way’ while others ‘continue the same way’ [41].

### 4.1. Policy Implications

The key findings of this study—showing that reinforcement of perceived health benefits information at the POP can increase women’s preferences for nutritional supplement products containing higher levels of folate and, in some cases, iodine—suggest a need for simultaneous strategies that (1) provide health benefits information prior to purchase (e.g., by targeting antenatal care services, public health initiatives, and other trusted sources of nutrition information), and (2) ensure health benefits information is available at the POP (e.g., by targeting supplement manufacturers and/or retailers).

#### 4.1.1. Creating Awareness Prior to Purchase (BEFORE entering the Shopping Environment)

Overall, 62% of women in the present sample received information about folate from their primary healthcare provider during pregnancy, and 32% received information about iodine, as reported in [27]. Other studies also report inconsistent provision of nutrition education during pregnancy [35,44,45]. Information received from healthcare providers can be an important source of motivation for women. Previous research shows that ‘If their doctor/health care provider advised them to do so’ was the most commonly identified motivating factor for taking a multivitamin containing folic acid daily [46]. Thus, informing women about the current supplement recommendations and benefits of supplementation during antenatal consultations should be standard practice to ensure all women of childbearing age or already pregnant are made aware of the benefits of folate and iodine before entering the shopping environment. This will require addressing the barriers to nutrition education and incorporating folic acid and iodine supplementation recommendations into standard practice. Healthcare providers perceive lack of time, resources, and relevant training as critical barriers to providing nutrition education [44]. Specific barriers to recommending iodine supplementation in pregnancy include awareness of the recommendation and the recommended dosage and duration [35].

Overall, the significant preference for products endorsed by a government science agency (Commonwealth Scientific and Industrial Research Organisation (CSIRO)) and a professional nutrition association (Dietitians Association of Australia (DAA)) in all segments in our study suggests the CSIRO, as well as dietitians and other nutrition experts, could also play an important role in promoting this information through mass-media and social-media channels that this population subgroup accesses. However, for segments not influenced by nutrient levels or information about health benefits of nutrients at the POP, strategies that do not rely on the individual taking an active role in decision-making may be more effective (e.g., direct recommendation or provision of appropriate products by trusted information sources) [14,47].

#### 4.1.2. Creating Awareness at POP

While there is room to improve information provision by healthcare providers and other trusted and qualified sources of nutrition information, product manufacturers also play an important role in enabling more informed consumer choices through the provision of health benefits information at the POP. Given that preconception and pregnancy are critical life stages during which maternal nutrition can impact infant health outcomes, a case can be made for stricter regulation of supplement products marketed for preconception and/or pregnancy. This is based on evidence that marketing supplement products as ‘preconception/pregnancy/lactation’ products can influence supplement use [12] and that the provision of health benefits information at POP increases the likelihood of choosing folate-containing products, at least among women with prior awareness of health benefits (reported here). Supplement manufacturers who market products for preconception and/or pregnancy would, therefore, appear to have a duty of care to either provide the recommended daily dosage of nutrients in the products and/or provide on-pack health benefits information to support informed choice.

The Therapeutic Goods Administration (TGA) regulates dietary supplements (vitamin and mineral products and non-prescription medicines) in Australia. Our results suggest that future reviews of labeling requirements undertaken by the TGA should consider additional changes that apply specifically to vitamin and mineral products marketed for preconception and/or pregnancy; these changes should aim to make it easier for consumers to identify and select products containing the recommended dosage of folate/iodine. Specific changes for consideration could include either a front-of-pack traffic light labeling system [48], signaling whether the product provides the recommended daily dosage of folate and iodine, or front-of-pack health benefits information for both folate and iodine (including information on the recommended duration of supplementation, to further guide appropriate use). In both cases (a traffic light labeling system and health benefits information), the regulation should also specify legibility criteria (e.g., minimum font size or surface area and contrast) and placement near the top of the label to increase visual saliency. These suggestions are based on eye-tracking research showing that: front-of-pack labels are most effective at drawing the attention of consumers [49]; food labels that are larger and more visually salient can increase attention capture, increasing the likelihood of choice [50,51]; and the traffic light format enables more efficient cognitive processing of nutrition information than either the nutrition table format or the guideline for daily amounts format [48]. Further, with increasing attention capture shown to increase the likelihood of choice, these label changes could also be effective at targeting consumers who do not appear to be motivated by health benefits information provided before or at POP.

### 4.2. Study Strengths and Limitations

A key strength of this study is the large sample size. Furthermore, unlike previous studies that looked at the effect of health/nutrition information on overall product preference, the present study examines the impact of health information on the importance of nutrient levels in the choice decision. Measuring the interaction between different combinations of prior and POP health information on the role of nutrient levels in the choice decision has not been done before, to our knowledge, and is key to understanding whether familiarity with health benefits can help women to make more informed purchase decisions based on nutrient levels. A limitation of this study is that we did not assess whether respondents visually attended to the health benefits information. This could be determined with eye-tracking technology in future research. Further, specific population subgroups may be underrepresented in our sample (e.g., women from certain cultural or ethnic backgrounds and with unplanned pregnancies), and the findings may not be generalizable to these subgroups. Lastly, random assignment to information conditions (showing or not showing health benefits information) was conditional on prior awareness of the health benefits. Given that the experimental conditions were not entirely randomly allocated, the interaction effects could be interpreted as a cross-sectional comparison of women with different levels of prior knowledge.

## 5. Conclusions

Here we show that the reinforcement of previously perceived health benefits information is most effective at increasing the importance pregnant women place on levels of folate (in all four segments) and iodine (in two segments, representing 63% of the sample) when choosing between different dietary supplement products. We further demonstrate that neither prior awareness of health benefits alone nor information provided at the POP without prior awareness is enough to increase the appeal of higher-folate/folate-containing products. This suggests that folate levels are not important in the choice decision for most women unless perceived health benefits information is reinforced at the POP. These findings can be valuable for informing the design of policies aimed at guiding women who are pregnant or planning pregnancy towards supplement products that will help to optimize maternal nutritional intake and infant growth and development.

## Figures and Tables

**Table 1:** Attributes and levels included in discrete choice experiment; health benefits of nutrients shown in the information treatments ^1^.

Attribute	Levels of Attributes
Specific product ^2^ (3 levels per alternative)	Fortified food: Yoghurt (1 tub/200 g), Bread (2 slices), Cereal (1 cup) Fortified drink: Juice (1 cup/250 mL), Milk (1 cup/250 mL), Water (1 cup/250 mL) Supplement tablet: Multivitamin tablet 1/day, Multivitamin tablet 2/day, Vitamin tablet 1/day
Folate (3 levels)	0, 400, 800 µg
Iodine (3 levels)	0, 150, 250 µg
Omega-3 (3 levels)	0, 115, 500 mg
Vitamin D (3 levels)	0, 200, 400 IU
Endorsement (6 levels)	Endorsed by the: (1) National Health and Medical Research Council (NHMRC); (2) Dietitians Association of Australia (DAA); (3) National Heart Foundation; (4) CSIRO (Commonwealth Scientific and Industrial Research Organisation). (5) Scientifically proven (6) No endorsement
Absorption (2 levels)	Easy to digest and absorb; No claim
Brand (2 levels)	A specific brand; No specific brand or a generic brand
Daily cost ($) ^3^ (3 levels per product)	Yoghurt: 0.90, 2.45, 4.00 Cereal: 0.25, 0.70, 1.20 Bread: 0.25, 0.75, 1.25 Juice: 0.30, 1.20, 2.20 Milk: 0.25, 0.90, 1.50 Water: 0.15, 1.20, 2.20 Multivitamin tablet (1/day): 0.25, 0.65, 1.10 Multivitamin tablet (2/day): 0.25, 0.65, 1.10 Vitamin tablet (1/day): 0.15, 0.35, 0.65
**Information treatments**	
**Nutrient**	**Health benefits information**
Folate	Adequate folate helps prevent neural tube defects such as spina bifida.
Iodine	Iodine plays an important role in the normal development of the baby’s brain.
Omega-3	Omega-3 fatty acids play an important role in the normal development of the baby’s brain and may help prevent premature birth and childhood allergy.
Vitamin D	Vitamin D plays an essential role in strengthening the baby’s bones.

^1^ Table adapted from [27]; ^2^ Alternative-specific attribute; ^3^ Product-specific attribute.

**Table 2:** Interaction effects showing the effect of information conditions (IC) on preferences for nutrient levels (*n* = 818).

	Parameter Estimates, β (SE)				Allocation to Each Information Condition	
	Segment 1 (20%)	Segment 2 (22%)	Segment 3 (43%)	Segment 4 (15%)	*n*	%
Folate IC *Folate (every 100 µg)						
**Not aware** of benefit, **Shown** information	0.009 (0.017)	−0.049 * (0.022)	0.023 (0.023)	−0.025 (0.033)	103	13%
**Not aware** of benefit, **Not shown** information	−0.007 (0.020)	0 (0.019)	−0.133 (0.024) ***	−0.049 (0.033)	102	12%
**Aware** of benefit, **Shown** information	0.033 (0.016) *	0.035 * (0.016)	0.091 (0.015) ***	0.055 * (0.022)	306	37%
**Aware** of benefit, **Not shown** information	−0.035 (0.015) *	0.014 (0.016)	0.020 (0.015)	0.019 (0.025)	307	38%
Iodine IC *Iodine (every 100 µg)						
**Not aware** of benefit, **Shown** information	−0.092 (0.049)	0.115 (0.054) *	0.079 (0.039) *	0.025 (0.07)	227	28%
**Not aware** of benefit, **Not shown** information	−0.084 (0.048)	−0.011 (0.049)	−0.213 (0.042) ***	−0.092 (0.065)	227	28%
**Aware** of benefit, **Shown** information	0.152 (0.070) *	−0.128 (0.062) *	0.183 (0.041) ***	0.138 (0.074)	182	22%
**Aware** of benefit, **Not shown** information	0.024 (0.064)	0.024 (0.059)	−0.049 (0.041)	−0.070 (0.079)	182	22%
Omega-3 IC *Omega-3 (every 100 mg)						
**Not aware** of benefit, **Shown** information	-0.024 (0.025)	0.0260 (0.025)	0.027 (0.019)	-0.025 (0.041)	158	19%
**Not aware** of benefit, **Not shown** information	−0.025 (0.026)	−0.020 (0.031)	−0.063 (0.019) ***	−0.061 (0.043)	157	19%
**Aware** of benefit, **Shown** information	0.039 (0.024)	−0.024 (0.026)	0.070 (0.016) ***	0.036 (0.032)	250	31%
**Aware** of benefit, **Not shown** information	0.010 (0.026)	0.018 (0.024)	−0.034 (0.016) *	0.050 (0.035)	253	31%
Vitamin D IC * Vitamin D (every 1 µg)						
**Not aware** of benefit, **Shown** information	−0.007 (0.012)	−0.003 (0.015)	0.059 (0.012) ***	0.016 (0.020)	173	21%
**Not aware** of benefit, **Not shown** information	−0.023 (0.013)	0.001 (0.013)	−0.058 (0.012) ***	−0.032 (0.018)	164	20%
**Aware** of benefit, **Shown** information	0.036 (0.012)	0.008 (0.013)	0.019 (0.010) *	0.007 (0.019)	240	29%
**Aware** of benefit, **Not shown** information	−0.006 (0.013)	−0.006 (0.012)	−0.021 (0.010) *	0.010 (0.020)	241	29%

* *p* < 0.05, *** *p* < 0.001. Standard errors are reported in brackets.

**Table 3:** Health benefits that participants believe are associated with each nutrient (*n* = 818).

	Folate	Iodine	Omega-3 Fatty Acids	Vitamin D
Important for baby’s brain development	29%	44%	56%	12%
Prevents neural tube defects such as spina bifida	75%	17%	8%	7%
Lowers risk of premature birth	15%	13%	7%	9%
Lowers risk of childhood allergy	5%	5%	10%	8%
Strengthens baby’s bones	14%	8%	12%	59%
Improves general health and well-being	23%	23%	39%	36%
No benefit	0%	1%	1%	0%
Don’t know	8%	31%	17%	17%

**Table 4:** Preference coefficients for the four segments of pregnant women (*n* = 818).

	Parameter Estimates, β Coefficient (Standard Error, SE)			
	Segment 1 (20%)	Segment 2 (22%)	Segment 3 (43%)	Segment 4 (15%)
Utility function				
Alternative ^1^				
Fortified food	−10.831 (4.947) *	−9.756 (4.947) *	−14.140 (4.948) **	−11.571 (4.948) *
Fortified drink	−9.932 (4.947) *	−10.799 (4.947) *	−14.145 (4.947) **	−11.766 (4.949) *
Supplement tablet	−11.662 (4.948) *	−12.960 (4.949) *	−14.180 (4.948) **	−10.072 (4.949) *
None	0.000	0.000	0.000	0.000
Folate (every 100 µg)	0.006 (0.018)	0.014 (0.019)	0.255 (0.020) ***	0.029 (0.029)
Iodine (every 100 µg)	0.026 (0.057)	−0.041 (0.059)	0.503 (0.053) ***	0.043 (0.080)
Omega-3 fatty acids (every 100 mg)	0.030 (0.015) *	0.038 (0.015) *	0.251 (0.013) ***	0.013 (0.023)
Vitamin D (every 1 µg)	0.025 (0.007) **	0.023 (0.008) **	0.107 (0.007) ***	0.055 (0.011) ***
Folate (every 100 µg) * Iodine (every 100 µg)	0.035 (0.017) *	0.020 (0.019)	0.010 (0.014)	0.033 (0.025)
Endorsement: fortified foods				
No endorsement	−0.273 (0.314)	−0.544 (0.133) ***	−0.320 (0.175) *	−0.195 (0.144)
Endorsed by the DAA	−0.228 (0.666)	0.645 (0.096) ***	0.123 (0.160)	0.028 (0.128)
Endorsed by the NHMRC	0.178 (0.480)	−0.101 (0.114)	−0.062 (0.205)	0.060 (0.156)
Endorsed by the National Heart Foundation	−0.071 (0.267)	−0.532 (0.085) ***	0.091 (0.119)	0.098 (0.113)
Endorsed by the CSIRO	0.356 (0.181) **	0.662 (0.074) ***	0.275 (0.12) **	0.195 (0.132)
Scientifically proven	0.037 (0.25)	−0.131 (0.086)	−0.107 (0.108)	−0.186 (0.111) *
Endorsement: fortified beverages				
No endorsement	−0.494 (0.271) *	−0.305 (0.112) ***	0.067 (0.161)	−0.181 (0.163)
Endorsed by the DAA	0.350 (0.184) *	0.503 (0.112) ***	−0.164 (0.116)	0.238 (0.138) *
Endorsed by the NHMRC	0.068 (0.334)	−0.339 (0.130) ***	−0.086 (0.151)	0.086 (0.172)
Endorsed by the National Heart Foundation	0.179 (0.182)	−0.326 (0.086) ***	−0.090 (0.101)	−0.039 (0.131)
Endorsed by the CSIRO	−0.079 (0.201)	0.504 (0.073) ***	0.324 (0.099) ***	0.028 (0.107)
Scientifically proven	−0.024 (0.260)	−0.036 (0.090)	−0.051 (0.097)	−0.133 (0.137)
Endorsement: supplement tablets				
No endorsement	−0.466 (0.292)	−0.580 (0.117) ***	−0.110 (0.191)	−0.573 (0.623)
Endorsed by the DAA	0.186 (0.194)	0.352 (0.097) ***	0.092 (0.169)	0.052 (0.346)
Endorsed by the NHMRC	−0.214 (0.270)	−0.007 (0.121)	0.286 (0.171) *	0.398 (0.317)
Endorsed by the National Heart Foundation	−0.045 (0.185)	−0.330 (0.086) ***	0.169 (0.161)	0.096 (0.279)
Endorsed by the CSIRO	0.428 (0.142) ***	0.616 (0.077) ***	−0.407 (0.229) *	0.139 (0.182
Scientifically proven	0.110 (0.151)	−0.050 (0.084)	−0.029 (0.111)	−0.112 (0.283)
Brand				
A specific brand	−0.090 (0.060)	0.035 (0.023)	−0.002 (0.035)	−0.011 (0.032)
No specific brand or a generic brand	0.090 (0.060)	−0.035 (0.023)	0.002 (0.035)	0.011 (0.032)
Absorption				
No claim	0.017 (0.078)	−0.085 (0.026) ***	−0.034 (0.046)	−0.031 (0.035)
Easy to digest and absorb	−0.017 (0.078)	0.085 (0.026) ***	0.034 (0.046)	0.031 (0.035)
Specific product: fortified foods				
Yoghurt (1 tub, 200 g)	−0.003 (0.482)	−0.250 (0.115) **	0.194 (0.258)	0.278 (0.355)
Bread (2 slices)	0.672 (0.216) ***	0.143 (0.111)	0.146 (0.219)	0.031 (0.250)
Cereal (1 cup)	−0.669 (0.400) *	0.106 (0.099)	−0.340 (0.169) **	−0.308 (0.202)
Specific product: fortified beverages				
Juice (1 cup, 250 mL)	−0.091 (0.288)	−0.096 (0.111)	0.398 (0.237) *	−0.163 (0.229)
Milk (1 cup, 250 mL)	−0.099 (0.252)	0.242 (0.111) **	−0.320 (0.215)	0.307 (0.198)
Water (1 cup, 250 mL)	0.190 (0.240)	−0.146 (0.103)	−0.077 (0.149)	−0.144 (0.177)
Specific product: tablets				
Multivitamin tablet ^1^ (1 per day)	−0.028 (0.310)	0.342 (0.116) ***	0.137 (0.172)	0.213 (0.548)
Multivitamin tablet (2 per day)	−0.074 (0.190)	−0.221 (0.093) **	−0.333 (0.194) *	−0.338 (0.617)
Vitamin tablet (1 per day)	0.102 (0.182)	−0.121 (0.090)	0.196 (0.149)	0.125 (0.273)
Price: fortified foods				
Yoghurt (1 tub, 200 g)	−0.249 (0.221)	0.026 (0.062)	−0.051 (0.085)	−0.129 (0.106)
Bread (2 slices)	−1.158 (0.385) ***	−0.728 (0.174) ***	−0.021 (0.288)	−0.029 (0.245)
Cereal (1 cup)	0.131 (0.202)	−0.043 (0.061)	0.138 (0.154)	0.058 (0.177)
Price: fortified beverages				
Juice (1 cup, 250 mL)	−0.316 (0.266)	−0.275 (0.082) ***	−0.166 (0.113)	0.053 (0.131)
Milk (1 cup, 250 mL)	−0.217 (0.208)	−0.064 (0.092)	−0.107 (0.132)	−0.076 (0.121)
Water (1 cup, 250 mL)	−0.215 (0.192)	−0.276 (0.067) ***	−0.021 (0.082)	−0.224 (0.085) ***
Price: tablets				
Multivitamin tablet ^2^ (1 per day)	0.378 (0.722)	−0.733 (0.232) ***	−0.121 (0.327)	0.328 (0.731)
Multivitamin tablet (2 per day)	−0.091 (0.327)	−0.220 (0.151)	0.056 (0.268)	0.513 (0.664)
Vitamin tablet (1 per day)	−0.476 (0.444)	0.349 (0.168) **	−0.233 (0.350)	−0.249 (0.431)
Class membership model				
Cohort				
South Australia	0.157 (0.076) *	−0.001 (0.072)	0.000 (0.060)	−0.156 (0.082)
National	−0.157 (0.076) *	0.001 (0.072)	0.000 (0.060)	0.156 (0.082)
University degree				
No	0.108 (0.075)	0.066 (0.071)	−0.060 (0.060)	−0.115 (0.082)
Yes	−0.108 (0.075)	−0.066 (0.071)	0.060 (0.060)	0.115 (0.082)
Intercepts of class membership model	−0.131 (0.083)	−0.045 (0.080)	0.612 (0.070) ***	−0.436 (0.102) ***

Abbreviations: NHMRC = National Health and Medical Research Council; DAA = Dietitians Association of Australia; CSIRO = Commonwealth Scientific and Industrial Research Organisation. * *p* < 0.05, ** *p* < 0.01, *** *p* < 0.001. Standard errors are reported in brackets. ^1^ Alternative variable is dummy coded such that the constant for the reference alternative (i.e., none) is equated to zero. Linear coding is used for price and the nutrients (folate, iodine, omega-3, vitamin D). Effects coding (where one level is treated as the reference and is equal to the negative sum of the coefficients for all other levels) is used for all other attributes. ^2^ ‘Multivitamin tablets’ contain additional vitamins and minerals whereas ‘vitamin tablets’ contain a single nutrient with no additional vitamins and minerals (this definition was provided to participants).

**Table 5:** Comparison of participant characteristics and behavior between segments (*n* = 818).

	Segment 1 (20%)	Segment 2 (22%)	Segment 3 (43%)	Segment 4 (15%)	Total (*n* = 818)	F/X^2^ Value ^1^	df ^2^	*p*-Value
Age, mean (SD)	29.5 (5.3) ^a^	31.3 (5.6) ^b^	31.0 (4.5) ^b^	33.2 (4.6) ^c^	31.1 (5.0)	13.63	3.337	<0.001
Body Mass Index (BMI), mean (SD)	24.4 (5.4)	24.4 (5.5)	25.2 (6.2)	26.1 (6.2)	25.0 (5.9)	2.44	3.814	0.063
Live in metropolitan area	77.4%	74.9%	79.8%	79.5%	78.2%	1.83	3	0.608
University educated	50.6%	50.9%	57.6%	59.8%	55.1%	4.60	3	0.204
Planned pregnancy	72.0%	73.1%	75.9%	75.4%	74.4%	1.16	3	0.762
Previous birth(s)	53.7% ^a,b,c^	60.8% ^c^	46.5% ^b^	61.5% ^a,c^	53.2%	13.79	3	0.003
Took supplements during this pregnancy	89.0% ^a,b^	84.8% ^b^	97.2% ^c^	95.1% ^a,c^	92.7%	30.86	3	0.000
Adhered to folic acid supplement recommendation	22.0%	22.4%	30.2%	30.6%	27.0%	6.48	3	0.090
Adhered to iodine supplement recommendation	17.3% ^a^	18.1% ^a,b^	25.5% ^a,b^	31.1% ^b^	23.2%	11.05	3	0.011

^a,b,c^ In each row, values followed by either no letter or the same letter are not statistically significantly different (5% level) based on results of post-hoc Tamhane’s T2 tests or comparison of column proportions using Bonferroni adjusted *p*-values. ^1^ F-value or Chi-squared value from overall significance test comparing the segments (using ANOVA or Pearson chi square test, respectively). ^2^ Values are the degrees of freedom from the ANOVA or Pearson chi square test.

## Data Availability

Data can be made available on request.

## Supplementary Materials

The following supporting information can be downloaded at: https://www.mdpi.com/article/10.3390/nu14091707/s1, Figure S1: Survey question assessing beliefs about health benefits of nutrients; Figure S2: Example of a choice set; Table S1: Model performance for models with up to six latent classes.
