# Supplementary material for: Testing the Impact of Familiarity with Health Benefits Information on Dietary Supplement Choice in Pregnancy: An Online Choice Experiment
Source: Nutrients. 2022 Apr 20;14(9):1707. doi: 10.3390/nu14091707 (PMC9105683; doi:10.3390/nu14091707)
Supplement: Supplementary file 1 [file nutrients-14-01707-s001.zip › nutrients-1662475-supplementary.pdf]

Online supplementary material

Figure S1. Survey question assessing beliefs about health benefits of nutrients.

16. There are various nutrients that might be added to food, drinks or vitamin tablets and which might be claimed to have health benefits. Some of these benefit an unborn child, some benefit the person who takes them, whilst others have no demonstrated clinical benefit. Please consider each nutrient below and tick the box(es) to indicate which health claim(s) you believe to be true.

(select all that apply for each nutrient; select at least one option in each row)

Nutrient	Important for baby's brain development	Prevents neural tube defects such as spina bifida	Lowers risk of premature birth	Lowers risk of childhood allergy	Strengthens baby's bones	Improves general health and well-being	No benefit	Don't know
Iodine	☐	☐	☐	☐	☐	☐	☐	☐
Folate	☐	☐	☐	☐	☐	☐	☐	☐
Omega-3 Fatty Acids	☐	☐	☐	☐	☐	☐	☐	☐
Vitamin D	☐	☐	☐	☐	☐	☐	☐	☐

Figure S2. Example of a choice set.

	Fortified food	Fortified drink	Supplement Tablet
**Product**	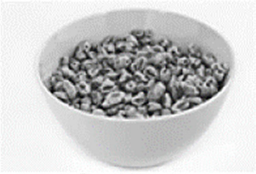 Cereal (1 cup)	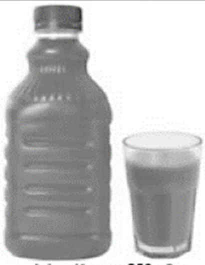 Juice (1 cup, 250ml)	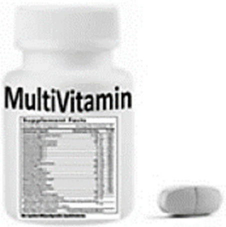 Multivitamin tablet (1 per day)
**Cost per day**	$0.25/day	$0.30/day	$0.65/day
**Brand**	A specific brand	A specific brand	No specific brand or a generic brand
**Endorsement**	Endorsed by the National Health and Medical Research Council (NHMRC)	Scientifically proven	Endorsed by the CSIRO
**Absorption**	No claim	No claim	Easy to digest and absorb
**Iodine**	-	150 µg	250 µg
**Folate**	800 µg	800 µg	400 µg
**Omega 3**	115 mg	-	500 mg
**Vitamin D**	400 IU	200 IU	-

If you want to review the above attributes again, please click here to open a separate glossary page.

Below are some information on nutrient benefits

Iodine plays an important role in the normal development of the baby's brain.

Vitamin D plays an essential role in strengthening baby's bones.

Q1 Which product do you like most? (choose one answer)

○ Fortified food ○ Fortified drink ○ Supplement tablet

Q2 Which product do you like least? (choose one answer)

○ Fortified food ○ Fortified drink ○ Supplement tablet

Q3 Would you actually buy your most preferred product? (choose one answer)

○ Yes ○ No

Table S1. Model performance for models with up to six latent classes.

Model	BIC	AIC	AIC3	CAIC	Number of parameters	L^2	Classification Error
1-class	24993.4	24748.7	24800.7	25045.4	52	24600.3	0.000
2-class	22659.9	22156.2	22263.2	22766.9	107	21897.9	0.039
3-class	21854.8	21092.2	21254.2	22016.8	162	20723.9	0.058
4-class	21552.1	20530.7	20747.7	21769.1	217	20052.3	0.071
5-class	21559.6	20279.4	20551.4	21831.6	272	19691.0	0.077
6-class	21651.1	20111.9	20438.9	21978.1	327	19413.6	0.109
